# Automated grading using natural language processing and semantic analysis

**DOI:** 10.1016/j.mex.2025.103395

**Published:** 2025-05-26

**Authors:** Ahmad Ayaan, Kok-Why Ng

**Affiliations:** Faculty of Computing and Informatics, Multimedia University, Cyberjaya, 63100, Selangor, Malaysia

**Keywords:** Natural language processing, Semantic analysis, Automatic Grading System, Automated Grading using Natural Language Processing and Semantic Analysis

## Abstract

Educational institutions' grading systems have evolved significantly toward automation, propelled by advances in Natural Language Processing (NLP) and Artificial Intelligence (AI). This research comprehensively explores automated grading systems, analyzing traditional methods alongside contemporary approaches and research on exam grading. Through extensive literature review, we examine the advantages and disadvantages of keyword-centric techniques, NLP-based systems, and hybrid models. We implement a novel NLP-based automatic grading system that combines multiple similarity measures with semantic analysis using TensorFlow's Universal Sentence Encoder. The system evaluates student responses by comparing them to reference answers using a weighted combination of edit similarity, cosine similarity, Jaccard similarity, normalized word count, and semantic similarity. Experiments conducted on 14 student responses demonstrate the system's ability to provide consistent and accurate grading while identifying cases requiring further human review. This study contributes to understanding automated grading systems, offering insights into their efficacy, limitations, and prospects in educational assessment practices.

• Hybrid evaluation: The proposed methodology combines traditional NLP techniques with advanced semantic analysis to provide comprehensive evaluation of student answers; the system integrates both surface-level textual similarity and deep semantic analysis to evaluate open-ended student responses.
• Weighted scoring: The system computes a weighted base score by combining four NLP metrics (Jaccard, edit distance, cosine similarity, normalized word count) and then blends this with a semantic similarity score from the Universal Sentence Encoder to assign marks.
• Rule-based final scoring: The final scoring layer applies threshold logic to assign zero, partial, or full marks based on semantic score and word count, and flags responses that fall into ambiguous ranges for teacher review.

## Graphical abstract

**Figure:**
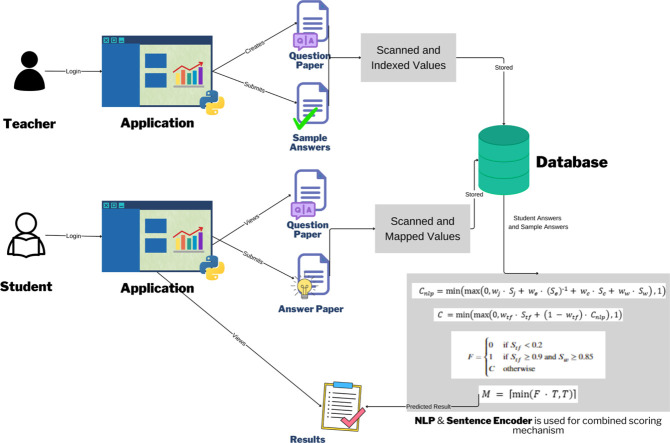

Specifications table

**Table:** 

Subject area:	Computer Science
More specific subject area:	Automatic Exam Paper Grading System
Name of your method:	Automated Grading using Natural Language Processing and Semantic Analysis.
Name and reference of original method:	None
Resource availability:	None

## Background

The manual grading of test papers is a labor-intensive and error-prone process, imposing a significant workload on educators. The inherent inconsistencies in human grading and the growing volume of examinations highlight the need for an efficient and accurate grading solution. This research aims to develop an automated exam paper grading system that enhances the grading process by offering improved efficiency and consistency. This software solution has the potential to transform the grading landscape, providing educators with a tool to define reference answers and bridge the gap between traditional manual grading and modern automation.

Rokade et al [1] criticize keyword-centric grading systems for favoring students who use specific terms, promoting rote learning rather than conceptual understanding. Ghosh and Fatima [2] propose an AEG framework tailored for the Indian context, integrating local language engines to handle language variations. Dadi and Ramesh [3] discuss AES systems and the challenges of evaluating content relevance, coherence, and adversarial inputs, while Das et al. [4] introduce a model employing LSTM and Named Entity Recognition to assess grammar, structure, and factual accuracy.

Rajesh and Kanimozhi [5] present an OCR and NLP-based system that converts handwritten papers to editable text for keyword-based grading, and Sharma and Jayagopi [6] combine handwriting recognition with AES, achieving high accuracy. Payak et al. [7] focuses on text summarization and keyword extraction, while Pathak et al. [8] describe machine learning-based essay scoring using grammar and content analysis.

Other research includes Sarker et al.’s INCITE system [9], which evaluates medical exams through NLP and achieves high accuracy, and Agarwal et al.’s AutoEval system [10] which uses TF-IDF and tokenization to streamline subjective exam grading. Sadhuram and Soni [11] develop a factoid QA system with machine learning to evaluate meaning, achieving reasonable accuracy, while Ibrahim et al. [12] employ LSTM models to improve grading efficiency and accuracy.

Süzen et al. [13] explore both supervised and unsupervised learning for grading UK GCSE exams, highlighting the potential of k-means clustering and training sets for accurate predictions. Guo et al [14] propose a knowledge integration approach for NLP tasks, using graph neural networks to enhance performance, while Xu [15] addresses computational fairness in recommender systems through advanced NLP and sequential models.

This project leverages Natural Language Processing (NLP) techniques to streamline the grading process. Furthermore, the TensorFlow Sentence Encoder model is employed to comprehend the semantics of student answers, facilitating a more accurate comparison with teacher-defined reference answers. This automated grading system is designed to expedite the grading process by automatically evaluating student responses against the provided reference answers, thus offering a reliable and consistent grading solution.

### Method details

The proposed methodology for evaluating student answers against reference answers is based on a combination of similarity measures, providing a comprehensive and nuanced assessment of students’ responses. Each of the similar measures has a weight that contributes to the calculations.

**Fig. 1:**
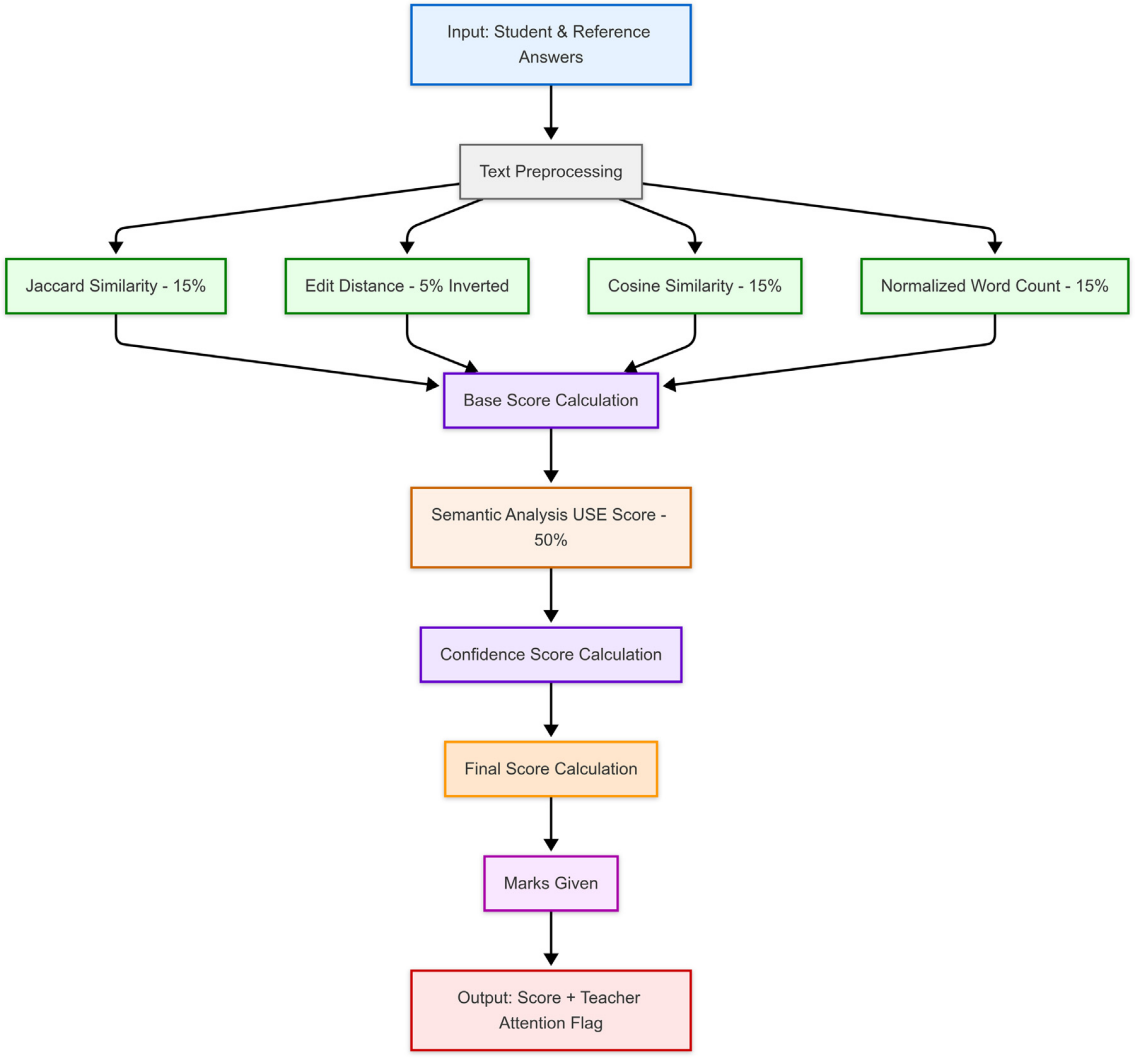
Overall architecture of the automated grading pipeline.

Fig. 1. illustrates the overall flow of the grading pipeline, from input preprocessing to final score computation using combined NLP and semantic similarity.

1. Natural Language Processing Technique:

The grading function utilizes multiple NLP techniques to evaluate the similarity between student answers and reference answers. These techniques capture various aspects of textual similarity, ensuring a thorough assessment. The primary NLP techniques used include:

a. Edit Similarity: Measures character-level changes needed to transform one text into another. This score captures the exactness of the student's answer, allowing for minor typographical errors while highlighting significant deviations. Lower edit distance indicates higher similarity. The edit distance score is assigned a weight of 0.05, out of 1, to ensure a balanced consideration of character-level differences in the assessment process. The edit similarity score is inverted while calculating, so the final score recorded is out of 1.
b. Cosine Similarity: Calculates the cosine of the angle between two vectors, evaluating the semantic closeness of word usage and frequency. This measure recognizes synonymous terms and different phrasing, supporting a fair and flexible grading process. The cosine similarity score, with a weight of 0.15 out of 1, captures semantics by robustly measuring the angle between the vectors of word frequencies.
c. Jaccard Similarity: Compares the intersection and union of keyword sets from student and sample answers. This metric assesses the presence of key terms and concepts, enhancing the system's ability to recognize partially correct answers using varied terminology. The Jaccard score, weighed 0.15 out of 1, respectively, offers insights into lexical similarity and synonym overlaps of keywords, providing a fairer assessment.
d. Normalized Word Count: Normalizes the student's word count against the reference answer's word count to ensure length variations do not disproportionately affect grading. It is calculated by dividing the sample answer's keywords by the student's answer's keywords. The normalized word count score is allotted a weight of 0.15, balancing its contribution in normalizing the impact of answer length across different metrics.

2. TensorFlow's Sentence Encoder:

The grading system integrates TensorFlow's Universal Sentence Encoder to evaluate the semantic similarity between student and reference answers. This approach provides a deep understanding of the context and meaning behind the responses.

a. Semantic Similarity (Universal Sentence Encoder): A significant part of the grading function is the semantic similarity score calculated using TensorFlow's Universal Sentence Encoder model. This score evaluates the meaning of the entire sentence, providing a deep understanding of the context and semantics of the student's answer compared to the sample answers provided by teachers.

The sentence encoder model delves into the deeper meaning and context of the texts, often leveraging advanced NLP techniques and pre-trained models. The semantic similarity score is weighed significantly at 0.5 out of 1. This measure is vital for understanding the broader context and intent behind the student's answer, going beyond mere textual similarity. Capturing the semantic relationship between the students and the reference answers ensures that the grading system accurately assesses the true understanding and knowledge demonstrated by the student.

3. Combined Scoring Mechanism:

The final grading is determined by combining the various similarity measures using weighted sums.

All these scores are combined using a weighted sum. The combined NLP score includes Jaccard, edit, cosine, and normalized word count scores, ensuring a balanced assessment based on different similarity measures, which also facilitates partial scoring. Finally, the semantic similarity score, weighted significantly from the TensorFlow model, is combined with the NLP score to produce the final confidence score. By integrating multiple similarity measures, the system provides partial scoring that recognizes varying degrees of correctness and understanding. This approach ensures a balanced assessment based on different aspects of textual and semantic similarity.

a. Combined NLP Score or Base Score Calculation: This score combines the Jaccard, Edit, and Cosine scores, and Normalized word count using their respective weights.
  (a) $$C_{nlp}=\min\left(\max\left(0,\;w_{j}\cdot S_{j}+w_{e}\cdot {\left(S_{e}\right)}^{-1}+w_{c}\cdot S_{c}+w_{w}\cdot S_{w}\right),\;1\right)$$
  where:
  $C_{nlp}:\;Base\;Score$
  $w_{j},\;w_{e},\;w_{c},\;w_{w}:\;Weights\;for\;Jaccard,\;Edit,\;Cosine,\;and\;Normalized\;Word\;Count\;$
  $S_{j},\;S_{e},\;S_{c},\;S_{w}:\;Jaccard,\;Cosine,\;and\;Normalized\;Word\;Count\;Scores\;$
  ${\left(S_{e}\right)}^{-1}:\;Inverse\;of\;the\;Edit\;Similarity\;Score$
b. Confidence Score Calculation: This score integrates the semantic similarity score obtained from the TensorFlow universal sentence encoder model with the combined NLP score.
  (b) $$C=\min\left(\max\left(0,\;w_{tf}\cdot \;S_{tf}+\;\left(1\;-\;w_{tf}\right)\cdot \;C_{nlp}\right),\;1\right)$$
  where:
  C:ConfidenceScore
  $w_{tf}:\;Weight\;for\;Semantic\;Similarity\;Score$
  $S_{tf}:\;Semantic\;Similarity\;Score$
  $C_{nlp}:\;Combined\;NLP\;Score\;from\;Equation\;\left(a\right)$
c. Final Score Calculation: If the semantic similarity score is very low (0.2), the final score is set to 0. If the semantic similarity score is very high (0.9) and the normalized word count is also high (0.85), the final score is set to 1. Otherwise, the final score is the confidence score, which facilitates partial scoring.
  (c) $$\begin{array}{ccc} F\; & = & \;\{0,\;if\;S_{tf}<\;0.2\\ & & 1,\;if\;S_{tf}\geq \;0.9\;and\;S_{w}\;\geq \;0.85\\ & & C,\;otherwise\\ & & \}\; \end{array}$$
  where:
  F:FinalScore
  $S_{tf}:\;Semantic\;Similarity\;Score$
  $S_{w}:\;Normalized\;Word\;Count\;Score$
d. Marks Given: The final score is scaled to the total possible marks and rounded to the nearest ceiling integer.
  (d) M=⌈min(F·T,T)⌉
  where:
  M:MarksGiven
  T:TotalMarksoftheQuestion

**Software and Environment:**

The proposed automated grading system was implemented using a combination of modern NLP libraries and deep learning frameworks. This section outlines the technical specifications of the implementation to enhance reproducibility.

Software Environment

• Programming Language: Python 3.8.10
• Operating System: Windows 11
• Development Environment: Jupyter Notebook 6.4.3

Libraries and Frameworks

• TensorFlow(Version 2.13.0): Used for implementing the Universal Sentence Encoder model
• NLTK(Version 3.8.1): Used for text preprocessing, tokenization, and basic NLP operations
• scikit-learn(Version 1.3.2): Used for implementing cosine similarity and other vector-based metrics
• NumPy(Version 1.24.3): Used for numerical operations and array manipulations
• Pandas(Version 2.0.3): Used for data handling and organization
• Matplotlib: (Version 3.5.0) and Seaborn (Version 0.11.2): Used for visualization, including radar charts

Model Specifications

Universal Sentence Encoder:

• Model: Universal-Sentence-Encoder/4 from TensorFlow Hub
• Embedding Dimension: 512
• Architecture: Transformer-based
• Trained on a variety of internet sources, including Wikipedia, news, web forums, and other web sources
• Default parameters were used without fine-tuning

### Method validation

Experiment:

This quiz forms part of a research experiment designed to evaluate the performance of an automated grading system tailored for educational purposes was created using Microsoft Forms. 14 students who took part in the assessment were asked to provide answers in a free-form format to simulate real-world scenarios where answers may vary in structure and detail. The primary goal was to test the system's capability to handle diverse responses and accurately assess the depth of understanding demonstrated by each participant.

**Fig. 2:**
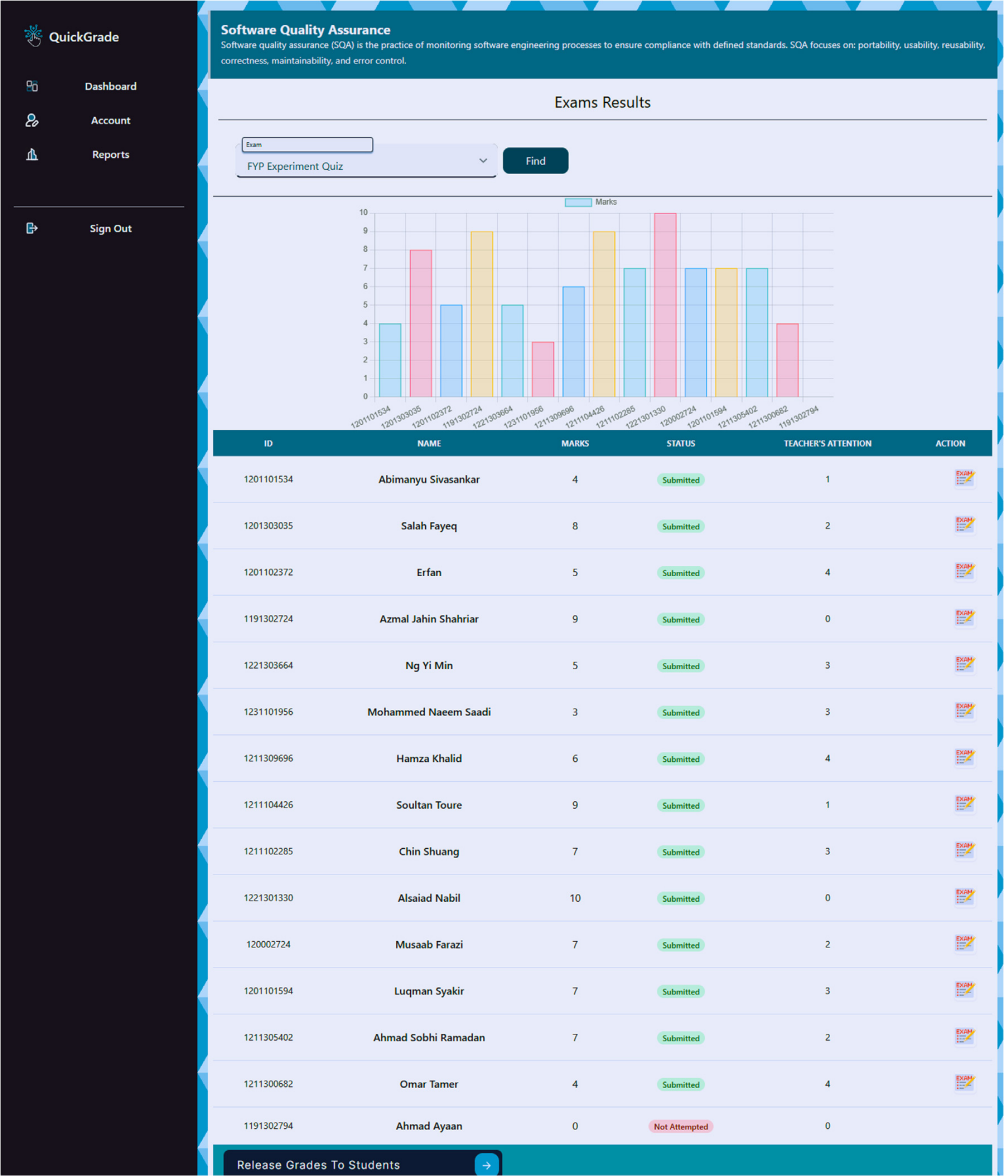
Results of the automated grading system applied to 14 student responses, showing the correlation between computed scores and the teacher’s attention.

Fig. 2. Results summary of the automated grading system. The table displays student IDs, names, and their respective scores, along with a “Teacher’s Attention” flag. This table also includes a "Teacher's Attention" column, indicating whether a teacher's review is required to finalize the score. This feature ensures that any anomalies or uncertainties in the automated grading process are flagged for further human review, maintaining the accuracy and fairness of the grading system.

Performance Comparison:

The results were obtained from different sample references and student answers. To further visualize and compare these results, we employ radar charts. Radar charts capture multidimensional data on the answer's similarity based on criteria such as Jaccard similarity, edit distance, cosine similarity, and semantic relevance.

**Fig. 3:**
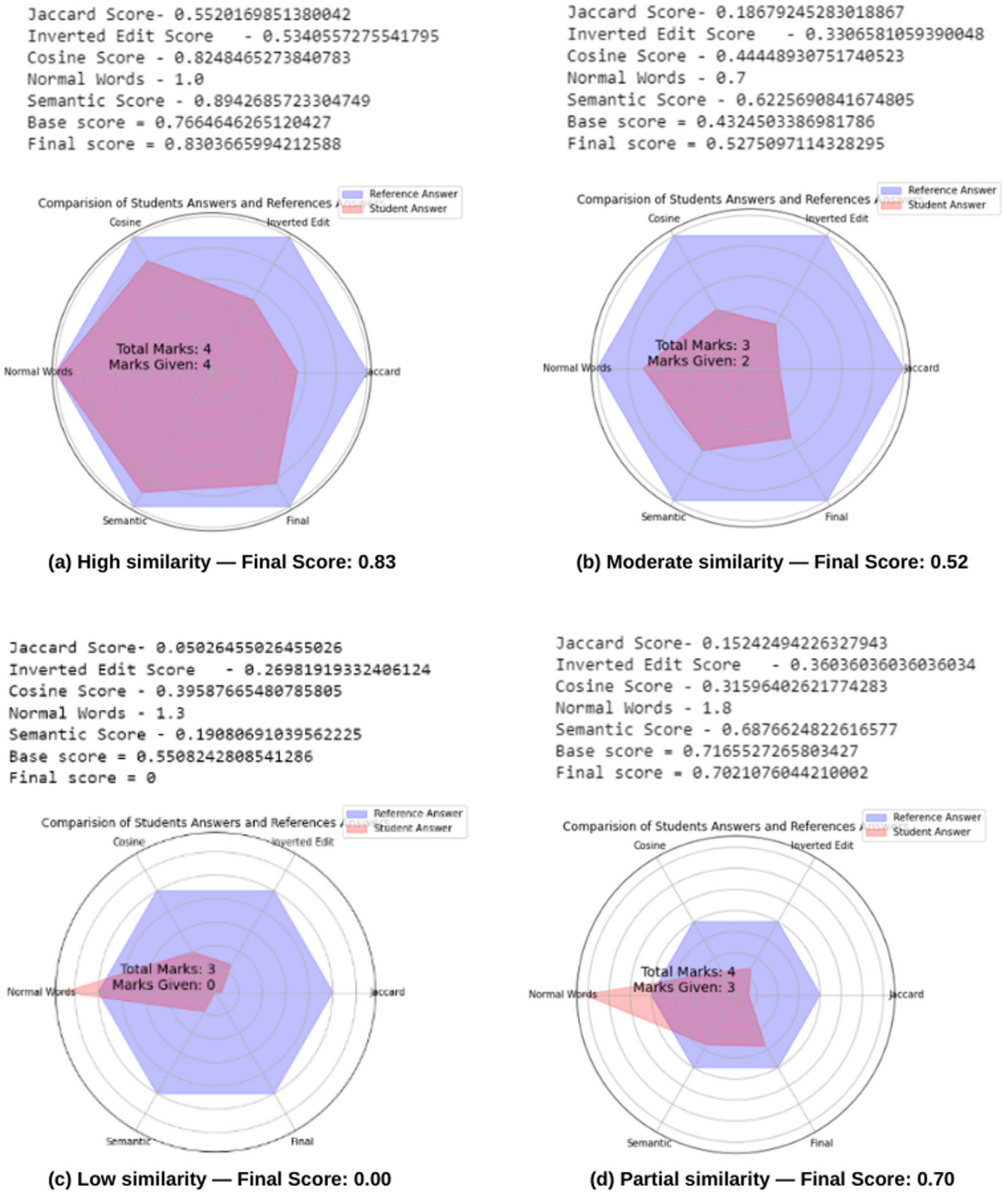
(a-d): Radar charts visualize multi-dimensional similarity analyses between student responses and reference answers. Each axis represents a distinct similarity metric: (a) high overall similarity with semantic score of 0.83 resulting in full marks; (b) moderate similarity with noticeable metric variations and semantic score of 0.52 enabling partial scoring; (c) low similarity despite higher word count, resulting in zero score; and (d) moderate overall similarity with semantic score of 0.70 and partial scoring despite extended response length.

Fig. 3a. Radar chart highlighting a significant overlap between the reference and student answers, indicating a high level of similarity across all evaluated metrics. Consequently, the Final score is determined as 0.83 because the Semantic score and Normal word count place it within the second criteria, and the student is awarded full marks for the answer.

Fig. 3b. Radar chart revealing discrepancies between the reference and student responses, with notable deviations in certain metrics such as edit distance and semantic relevance. Here, the Final score is determined as 0.52 because the Semantic score is not higher than 0.9, making partial scoring feasible.

Fig. 3c. Radar chart demonstrates low similarity across most metrics despite a higher word count. Despite the higher word count, the final score was determined to be 0. This indicates that the student's answer had significant deviations from the reference answer across several metrics, resulting in a low semantic score and an overall low final score.

Fig. 3d. Radar chart for a case with moderate overall similarity. The Final score is determined as 0.70 because the Semantic score is also not high enough for this case, making partial scoring feasible, despite the longer word count.

### Limitations

Despite demonstrating considerable success in automated grading, the system exhibits specific limitations worth addressing. First, it struggles with highly subjective or creative responses that demand nuanced context awareness beyond current NLP capabilities. Second, the system's reliance on digital text input presents challenges for implementation in traditional educational settings where handwritten examinations remain prevalent. Third, while the semantic analysis component significantly improves understanding, it may still miss cultural or domain-specific references that human graders would recognize. These limitations define clear trajectories for future research and development.

## CRediT authorship contribution statement

**Ahmad Ayaan:** Conceptualization, Methodology, Software, Visualization, Validation. **Kok-Why Ng:** Supervision, Writing – review & editing.

## Declaration of competing interest

The authors declare that they have no known competing financial interests or personal relationships that could have appeared to influence the work reported in this paper.

## Data Availability

The data that has been used is confidential.
